# Supplementary material for: PM2.5-Associated Hospitalization Risk of Cardiovascular Diseases in Wuhan: Cases Alleviated by Residential Greenness
Source: Int J Environ Res Public Health. 2022 Dec 31;20(1):746. doi: 10.3390/ijerph20010746 (PMC9819994; doi:10.3390/ijerph20010746)
Supplement: Supplementary file 1 [file ijerph-20-00746-s001.zip › ijerph-2095781-supplementary.pdf]

Supplementary material

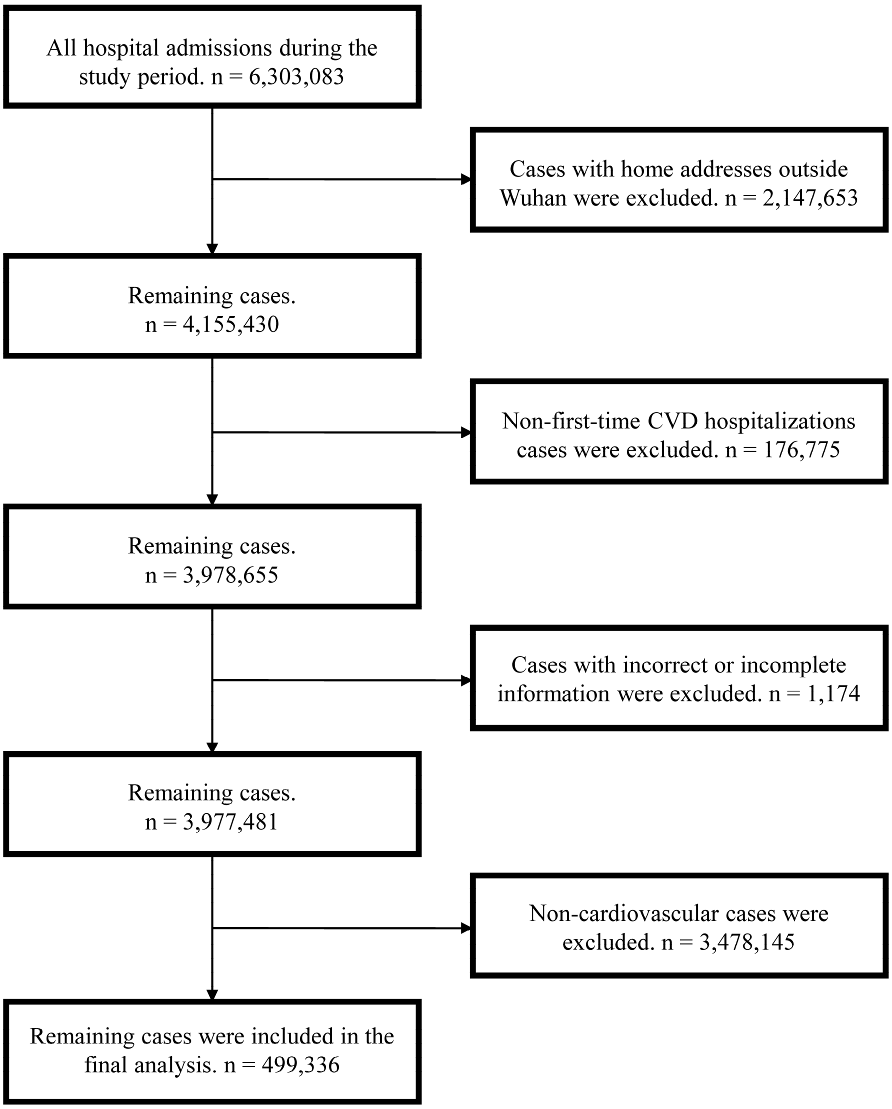

Figure S1. Flowchart of the selection process for the study population

Table S1 Distribution of exposure to fine particulate ($PM_{2.5}$) matter and natural conditions on case days

Variables	Mean ± SD	Min	P_{25}	P_{50}	P_{75}	Max
Pollution						
$PM_{2.5}$ (μg/m^3) on the admission day	44.8 ± 27.8	3	24.8	38.3	56.3	267.7
$PM_{2.5}$ (μg/m^3) in the year before admission	46.9 ± 4.7	37.9	45	47.1	50.1	62.7
Natural conditions						
Temperature (°C)	17.5 ± 9.2	−3.8	9.4	17.5	25.1	33.9
Humidity (%)	78.8 ± 10.2	41	72	79	86	100
NDVI within 250 m	0.46 ± 0.14	<0.01	0.36	0.42	0.52	0.99
NDVI within 500 m	0.46 ± 0.14	<0.01	0.38	0.43	0.52	0.93

Abbreviation: SD, standard deviation; Min, minimum; P_{25}, the 25th percentile; P_{50}, the 50th percentile; P_{75}, the 75th percentile; Max, maximum.

Table S2 ER% and 95% CI of hospitalization risk for cardiovascular diseases associated with every 10 μg/m3 increase in lag0-2 of short-term $PM_{2.5}$ using different df of natural cubic spline (NCS) function for the meteorological variables (3–6 df)

temperature df	humidity df	Total CVD	Hypertension	CHD	Stroke
3	3	0.78 (0.58, 0.99)	0.53 (0.03, 1.04)	1.01 (0.62, 1.39)	0.69 (0.32, 1.06)
4	4	0.64(0.44,0.84)	0.4(-0.11,0.91)	0.86(0.48,1.24)	0.57(0.2,0.94)
5	5	0.67(0.46,0.88)	0.44(-0.07,0.95)	0.9(0.51,1.28)	0.59(0.22,0.96)
6	6	0.7(0.49,0.9)	0.44(-0.07,0.95)	0.92(0.54,1.31)	0.63(0.26,1)
3	6	0.78(0.58,0.99)	0.52(0.01,1.02)	1.01(0.63,1.39)	0.7(0.33,1.06)
6	3	0.7(0.5,0.91)	0.47(-0.04,0.98)	0.92(0.54,1.31)	0.62(0.26,0.99)

Abbreviations: The statistically significant estimates are highlighted in bold. CHD, coronary heart disease. CVD, cardiovascular disease

Table S3 ER% and 95% CI of hospitalization risk for cardiovascular diseases associated with every 10 μg/m^3 increase in different lag days of $PM_{2.5}$ using CCO design*.

lag	Total CVD	Hypertension	CHD	stroke
lag0-3	**0.34(0.12,0.57)**	0.23(-0.34,0.8)	**0.62(0.21,1.04)**	-0.1(-0.51,0.3)
lag0-2	**0.6(0.38,0.81)**	0.52(-0.01,1.06)	**0.89(0.49,1.28)**	0.07(-0.31,0.46)
lag0-1	**0.68(0.48,0.88)**	**0.57(0.06,1.07)**	**0.96(0.6,1.33)**	0.23(-0.13,0.58)
lag3	-0.35(-0.52, -0.18)	-0.47(-0.89, -0.04)	-0.27(-0.58,0.05)	-0.38(-0.69, -0.08)
lag2	0.15(-0.02,0.32)	0.18(-0.26,0.61)	0.29(-0.03,0.61)	-0.2(-0.51,0.11)
lag1	**0.5(0.33,0.68)**	**0.48(0.04,0.92)**	**0.74(0.42,1.07)**	0.05(-0.27,0.36)
lag0	**0.59(0.41,0.77)**	0.43(-0.03,0.88)	**0.8(0.47,1.13)**	**0.32(0,0.64)**

Abbreviations: The statistically significant estimates are highlighted in bold. lag0-3, the moving average concentration on the present day and the previous 3 days. lag0-2, the moving average concentration on the present day and the previous 2 days. lag0-1, the moving average concentration on the present day and the previous 1 day.

* A symmetric **case-crossover design (days: ±7, 14)** was used to identified 499336 cases and 1997344 controls.

Table S4 ERs% and 95% CI of cardiovascular hospitalization per 10 μg/m^3 increase in 3-day moving average (lag 0–2) concentration of $PM_{2.5}$ in the low and high residential greenness areas divided by levels of greenness [a]

Admission	All	Low greenness	High greenness
Total CVD	0.78 (0.58, 0.99)	0.99 (0.70, 1.28)	0.56 (0.27, 0.86)
Hypertension	0.53 (0.03, 1.04)	0.91 (0.20, 1.62)	0.13 (−0.60, 0.85)
CHD	1.01 (0.62, 1.39)	1.32 (0.78, 1.86)	0.66 (0.12, 1.20)
Stroke	0.69 (0.32, 1.06)	0.75 (0.24, 1.26)	0.63 (0.11, 1.16)

[a] Low and high greenness were defined by the median of normalized difference vegetation index within 500 m.

Table S5 ERs% and 95% CI of hospitalization risk for cardiovascular diseases associated with every 10 μg/m^3 increase in short-term $PM_{2.5}$ at lag0-2: stratified by dual environmental factors

admissions	[a] Long-term $PM_{2.5}$ exposure	[b] NDVI			
		q1	q2	q3	q4
Total CVD	low	**1.45(0.76,2.15)**	**1.09(0.39,1.79)**	0.47(-0.23,1.17)	0.04(-0.53,0.61)
	high	**0.67(0.15,1.19)**	**1.4(0.89,1.92)**	**0.82(0.31,1.33)**	-0.12(-0.82,0.59)
Hypertension	low	**2.17(0.46,3.91)**	0.45(-1.24,2.17)	0.3(-1.41,2.04)	-0.2(-1.75,1.38)
	high	0.13(-1.11,1.38)	0.82(-0.41,2.06)	0.51(-0.68,1.73)	-0.71(-2.41,1.02)
CHD	low	**1.28(0.02,2.56)**	0.41(-0.87,1.71)	0.81(-0.45,2.1)	0.12(-1.03,1.28)
	high	**0.94(0,1.89)**	**2.19(1.26,3.13)**	**1.07(0.15,2)**	-0.18(-1.5,1.16)
Stroke	low	**1.11(0.05,2.19)**	0.5(-0.72,1.73)	0.49(-0.73,1.73)	0.29(-0.62,1.22)
	high	0.68(-0.26,1.62)	0.75(-0.19,1.69)	0.53(-0.43,1.49)	0.98(-0.28,2.26)

Abbreviations: The statistically significant estimates are highlighted in bold. CHD, coronary heart disease. CVD, cardiovascular disease. lag0-2, the moving average concentration on the present day and the previous 2 days.

[a] low and high long-term PM2.5 were divided by the median of all participants' long-term PM2.5 concentration **(6 months).**

[b] Divided into four parts based on the quartile of the all participants' **NDVI within 500 m**.

Table S6. ERs% and 95% CI of the cardiovascular hospitalizations per 10 μg/m^3 increase in the $PM_{2.5}$ at lag0-2 stratified by gender and age in the G_HE_L and the G_LE_H

Subgroup		Total CVD	Hypertension	CHD	Stroke
Male	[a] G_HE_L	0.31 (−0.29, 0.91)	−0.11 (−1.74, 1.54)	0.46 (−0.48, 1.41)	0.35 (−0.64, 1.35)
	G_LE_H	0.97 (0.48, 1.47)	0.3 (−0.94, 1.55)	1.7 (0.81, 2.59)	0.81 (−0.05, 1.68)
Female	G_HE_L	0.52 (−0.13, 1.17)	0.17 (−1.46, 1.82)	0.47 (−0.41, 1.36)	0.47 (−0.62, 1.57)
	G_LE_H	0.79 (0.23, 1.36)	0.88 (−0.39, 2.17)	1.25 (0.22, 2.28)	0.37 (−0.68, 1.44)
<45 years	G_HE_L	0.25 (−1.27, 1.8)	−0.98 (−4.38, 2.54)	1.46 (−1.37, 4.38)	0.02 (−3.71, 3.89)
	G_LE_H	0.83 (−0.5, 2.19)	1.72 (−0.52, 4.04)	1.66 (−1.15, 4.63)	−0.46 (−3.73, 2.93)
45–54 years	G_HE_L	0.57 (−0.64, 1.79)	1.93 (−0.87, 4.8)	0.27 (−2.17, 2.76)	−0.9 (−3.01, 1.25)
	G_LE_H	0.43 (−0.6, 1.46)	0.27 (−1.89, 2.48)	0.75 (−1.19, 2.72)	0.85 (−1.15, 2.9)
55–64 years	G_HE_L	−0.84 (−1.72, 0.05)	−0.5 (−2.84, 1.9)	−1.17 (−2.77, 0.45)	−1.17 (−2.63, 0.31)
	G_LE_H	1.29 (0.56, 2.02)	1.04 (−0.7, 2.81)	1.42 (0.37, 2.47)	1.71 (0.37, 3.06)

>64 years	G_HE_L	0.99 (0.39, 1.59)	−0.05 (−1.7, 1.64)	1.07 (−0.06, 2.21)	1.34 (0.38, 2.3)
	G_LE_H	0.91 (0.41, 1.41)	0.08 (−1.17, 1.35)	1.81 (0.93, 2.69)	0.38 (−0.46, 1.24)

Abbreviations: G_HE_L, high-greenness and long-term low-level PM2.5 exposure; G_LE_H, low-greenness and long-term high-level PM2.5 exposure.

[a] The G_HE_L and the G_LE_H were defined by the median of participants' **NDVI within 250m** and the median of long-term $PM_{2.5}$ concentration (**1 year**).

.

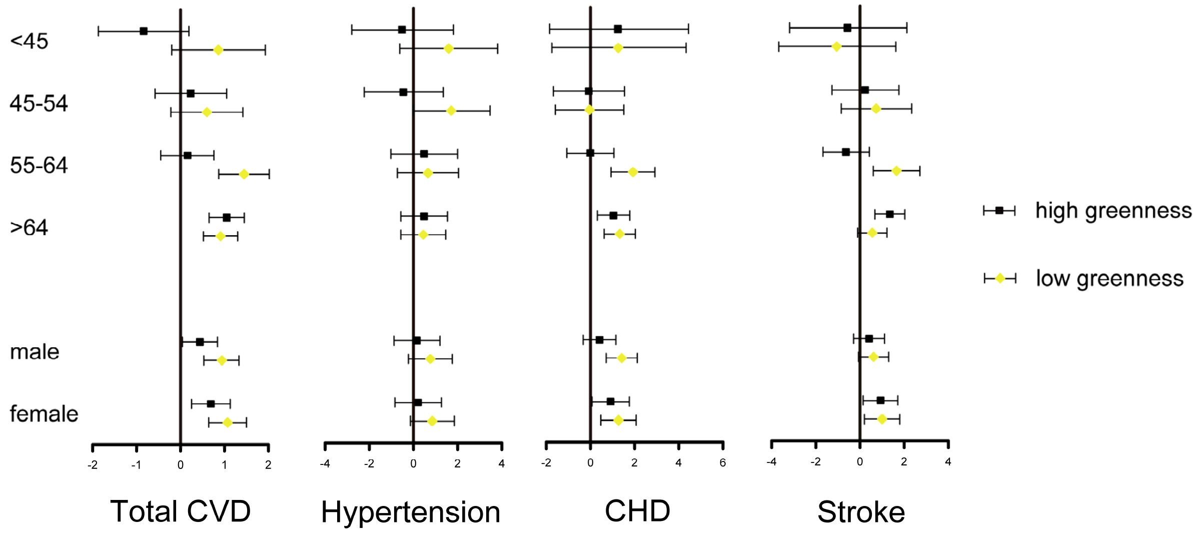

Figure S2. ER% (95% CI) of the CVD admissions per 10 μg/m^3 increase in the lag0–2 of $PM_{2.5}$ stratified by age and gender in high and low greenness areas.

We developed the LUR models using a range of geographic predictors, including types of land use (https://earthexplorer.usgs.gov/), the length of roads (http://www.openstreetmap.org), the nearest distance between the station and the road, the number of industrial sources (http://www.whepb.gov.cn/), population density (https://sedac.ciesin.columbia.edu/), and digital elevation (http://srtm.csi.cgiar.org). Considering the long span of our research period, we use the two-years average concentration for modeling to better assess the individual's exposure. Refer to the standardized procedure of the European Study of Cohorts for Air Pollution Effects (ESCAPE), the LUR model was developed based on some principles. Two LUR models were built respectively, each of which explained at least 78% of the variability of the monitor concentration at a fixed site, and yielded high leave-one-out-cross-validation (LOOCV) R^2 value.

Table S7 Description of Developed LUR models for $PM_{2.5}$ in different year.

Pollutant	Study period	* [a] LUR model	[b] R^2 of model	[c] R^2 validation
$PM_{2.5}$	2016.1.1–2017.12.31	41.03 + 0.32 × SECONDDISTINVNEARC2_500 + 0.2 × SECOND_1000 + 1.43×10^{-7} × RESINUM_3000	0.78	0.61
$PM_{2.5}$	2018.1.1–2019.12.31	46.52 + 0.49 × SECONDDISTINVNEARC2_500 – 1.96×10^{-7} × GL_3000	0.86	0.67

Abbreviation: RESINUM_3000: resident number in the 3,000-meter buffer zone; GL_3000: the area of green land within the 3,000-meter buffer zone; SECOND_1000: The total length of secondary highway within the 1,000-meter buffer zone; SECONDDISTINVNEARC2_500: inverse squared distance to the secondary highway within 500-meter buffer zone

[a] Some variables are buffers with _X indicating the radius of the buffer in meters.
[b] R^2 of model is a key indicator of model accuracy. For example, R^2=0.78 indicates the model explained 78% of the variability of $PM_{2.5}$ concentration of monitor stations.
[c] R^2 validation were derived from the procedure of leave-one-out-cross-validation (LOOCV), higher R^2 values indicates better predictive and extrapolation capabilities of the model.
* When constructing the multivariate linear model in the above table, we used the forward algorithm to develop a simpler model from a large number of predictor variables in order to maximize the interpretation of air pollution variability. The forward algorithm is used in the ESCAPE

(European Study of Cohorts to Air Pollution Effects) project of the European Union. The specific steps are as follows: (1) A unitary linear regression model between dependent variables and all predictive variables is constructed, and the model with the highest R^2 after adjustment is selected as the starting model. (2) Add variables to the regression equation gradually according to certain rules until the increase of model R^2 does not exceed 1% after the addition of variables. The rule of adding variables is: After adding variables, the symbols of all the coefficients of independent variables in the regression equation conform to the prior assumptions; (3) Finally, the variables with P value greater than 0.10 and variance inflation factor (VIF) higher than 3 are eliminated from the model. Leave-one-out-cross-validation (LOOCV) is to develop a model for *N-1* of these sites, then predict the concentration at the legacy site and compare the predicted concentration with the actual measured concentration at the legacy site. This process was repeated *N* times to test the predicted and observed concentrations at all sites, and then R^2 was used to describe the results of how good the model was.
